# Spinal aspergillosis: a rare complication of COVID-19 infection

**DOI:** 10.1186/s41984-021-00140-y

**Published:** 2022-01-27

**Authors:** Rumana Makhdoomi, Nayil Malik, Jagdish Charan, Azhar Malik, Sarbjit Singh

**Affiliations:** 1 Department of Pathology, Sheri-Kashmir-Institute of Medical Sciences, Srinagar, Kashmir 190011 India; 2 Department of Neurosurgery, Sheri-Kashmir-Institute of Medical Sciences, Srinagar, Kashmir 190011 India

**Keywords:** Spinal Aspergillosis, COVID-19, Fungal infection

## Abstract

**Background:**

Several complications have been reported in COVID-19 infection. Most of the complications include secondary infection.

**Case presentation:**

We report an 85-year-old male who presented with cauda equina syndrome 7-months after contracting COVID-19 infection. We excised an extradural mass which on examination proved to be Spinal Aspergillosis.

**Conclusions:**

Spinal Aspergillosis should be kept in mind in patients who present with local spinal pain with or without neurological deficit after COVID-19 infection.

## Background

Coronavirus infection (COVID-19) affects all organs, and secondary bacterial and fungal infections have been reported frequently [1]. However, vertebral osteomyelitis secondary to Aspergillus has not been reported so far. We report an elderly male who after recovery from COVID-19, developed lumbar spinal Aspergillosis with cauda equina syndrome.

## Case presentation

**Fig. 1:**
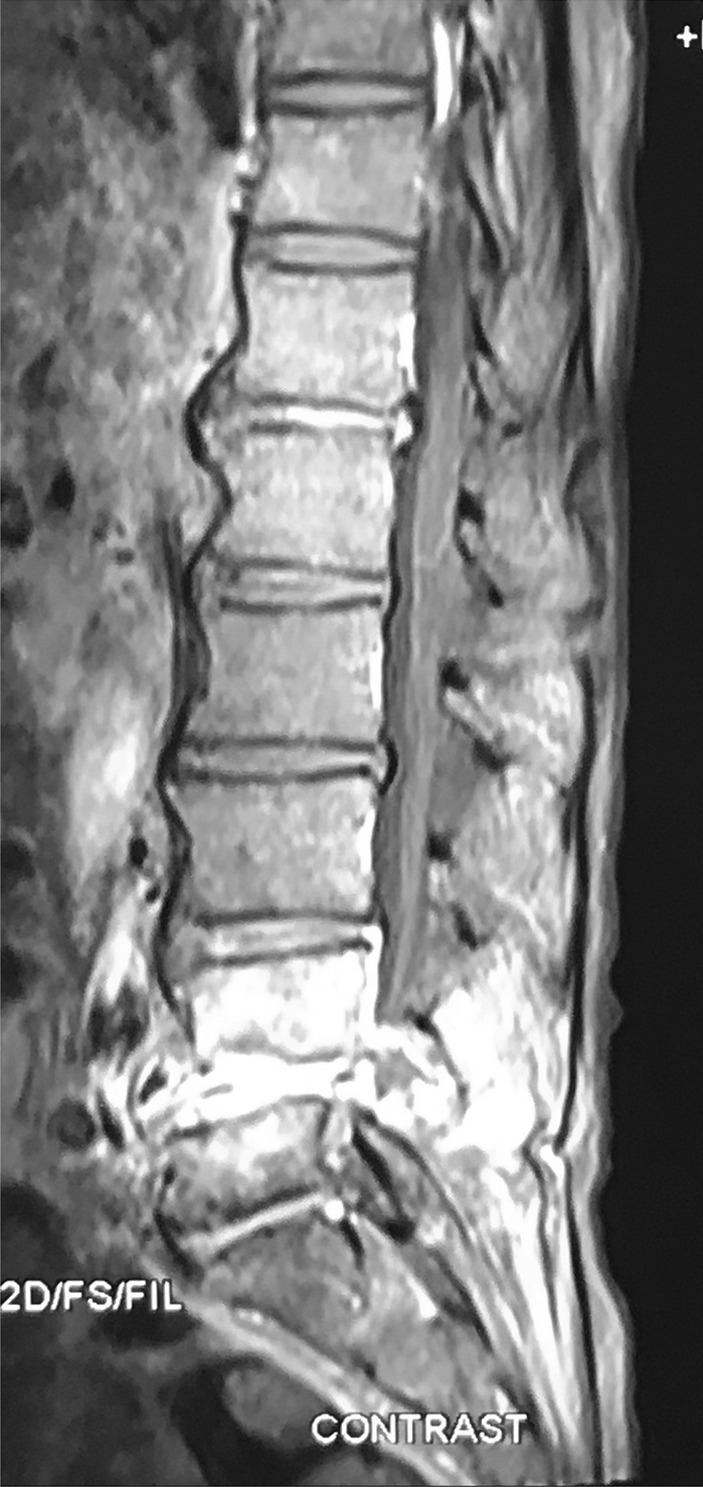
MRI lumbosacral spine sagittal contrast T1-weighted image shows avid contrast enhancement

**Fig. 2:**
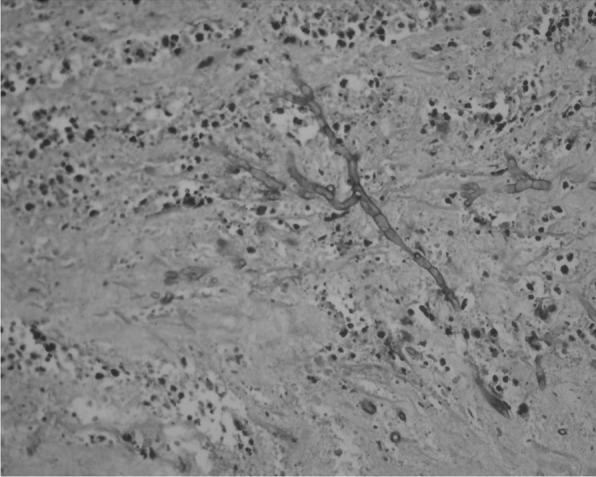
Periodic-Acid-Schiff (PAS) stain shows slender septate acute-angled branching hyphae of Aspergillus

An 85-yr-old male patient reported to Accident and Emergency with a history of low backache of 1-month duration and weakness of lower limbs with urinary retention of 2-days duration. His recent history revealed that he had moderate COVID-19 infection 7-months back for which he had received steroids. He was non-diabetic and was not on any immunosuppressive therapy. On examination, power was 3/5 power in lower limbs. Contrast MRI of the lumbosacral spine showed features of lumbar spondylodiscitis with epidural collection at L4-L5 with narrowing of canal diameter (Fig. 1). The patient was taken up for L4 and L5 laminectomy with debridement of the epidural soft tissue mass. The histopathology examination showed features of Aspergillosis (Fig. 2). The patient was administered parenteral liposomal amphotericin-B, and the patient is doing well on follow-up. His power in the lower limbs is normal at 3-months follow-up.

## Discussion

Spinal epidural abscess usually spreads from adjacent infective sites, such as spondylodiscitis, generally occurring in patients with diabetes, obesity, cancer, recreational intravenous drug abusers or immunocompromised patients. Bacterial epidural abscess is common, and a fungal abscess at this site is very rare [2]. From a database of 1,053 spinal epidural abscess patients, Yang et al. identified 9 patients with a fungal spinal epidural abscess of which seven patients had concurrent immunosuppression. Five of nine cases involved the thoracic spine, and eight had vertebral osteomyelitis. Aspergillus fumigatus was isolated from 2 (22%) patients, and Candida species were isolated from 7 (78%) patients [3].

Various case reports describing neuroimaging manifestations in patients with COVID-19 infection include demyelinating lesions, encephalitis and paraspinal myositis [4]. Talamonti et al. [5] reported 6 cases of bacterial spinal epidural abscess after COVID-19 infection.

Spinal Aspergillosis has been reported as a complication of HIV infection also [6]. However, it has not been reported as a complication in COVID-19 infection. Aspergillosis of the lung is a serious complication of COVID-19 patients that may not respond well to medical therapy. It has been seen that patients who have received corticosteroids as part of treatment for COVID-19 are susceptible to fungal infections [7]. Patients being treated with corticosteroids potentiate risk for Aspergillus infection by decreasing intracellular killing of spores by macrophages, thereby allowing intracellular germination [8]. All studies related to COVID-19 fungal infections report occurrence within 2-weeks after the appearance of COVID-19 symptoms [9]. Our patient was an 85-year-old patient, who was previously diagnosed with spinal Aspergillosis 7-months after COVID-19 infection. He had received Dexamethasone 6 mg OD for one week which was then tapered over the next 3 days as part of treatment for his COVID-19 infection. He presented to us with low backache and cauda equina syndrome. On imaging, the lesion had a focus of hypointense signal on T-2 weighted sequence. The reason for this hypointensity is supposed to be the presence of paramagnetic and ferromagnetic elements intrinsic to the fungi [10]. The lesion was enhancing avidly on contrast (Fig. 1). Laminectomy at L4 and L5 levels was done, and during surgery, we found an ash-grey avascular epidural mass which was removed. There was no abscess. Patient Histopathology revealed fibro-collagenous spinal tissue with extensive areas of necrosis infiltrated by abundant fungal hyphae. The fungal hyphae were parallel, narrow, septate with acute angulation. PAS (Periodic Acid Schiff) stain highlighted the hyphae, and confirmation was done on Gomori methenamine silver (GMS) stain (Fig. 2). The differential diagnosis in this situation would be Mucormycosis. However, mucor hyphae are broad, non-septate with right-angled branching pattern [11].

To our knowledge, this is the first case report of Aspergillosis of the spine as a complication of COVID-19 infection.

## Conclusions

Although various fungal infections of the lung are being readily diagnosed in patients who have received steroids as a part of treatment in COVID-19 infections, the diagnosis of spinal Aspergillosis should be kept in mind in patients who present with local pain with or without neuro deficit.

## Data Availability

Not applicable.

## Abbreviation

COVID: Corona virus disease

## References

[1] Jin Y, Yang H, Ji W, Wu W, Chen S, Zhang W, Duan G (2020). Virology, epidemiology, pathogenesis, and control of COVID-19. Viruses.

[2] Khursheed N, Dar S, Ramzan A, Fomda B, Humam N, Abrar W, Singh S, Sajad A, Mahek M, Yawar S (2017). Spinal epidural abscess: report on 27 cases. Surg Neurol Int.

[3] Yang H, Shah AA, Nelson SB, Schwab JH (2019). Fungal spinal epidural abscess: a case series of nine patients. Spine J.

[4] Mehan WA, Yoon BC, Lang M, Li MD, Rincon S, Buch K (2020). Paraspinal myositis in patients with COVID-19 infection. Am J Neuroradiol.

[5] Talamonti G, Colistra D, Crisà F, Cenzato M, Giorgi P, D'Aliberti G (2020). Spinal epidural abscess in COVID-19 patients. J Neurol.

[6] Murtagh RD, Post MJ, Bruce J, Post KK (2008). Spinal epidural aspergillosis in a patient with HIV resulting from long-standing (3 years) lung infection. Am J Neuroradiol.

[7] Singh AK, Singh R, Joshi SR, Misra A (2021). Mucormycosis in COVID-19: a systematic review of cases reported worldwide and in India. Diabetes Metab Syndr.

[8] Wagner DK, Varkey B, Sheth NK, DaMert GJ (1985). Epidural abscess, vertebral destruction, and paraplegia caused by extending infection from an aspergilloma. Am J Med.

[9] Bhatt K, Agolli A, Patel MH, Garimella R, Devi M, Garcia E, Amin H, Domingue C, Guerra Del Castillo R, Sanchez-Gonzalez M (2021). High mortality co-infections of COVID-19 patients: mucormycosis and other fungal infections. Discoveries (Craiova).

[10] Zinreich SJ, Kennedy DW, Malat J, Curtin HD, Epstein JI, Huff LC, Kumar AJ, Johns ME, Rosenbaum AE (1988). Fungal sinusitis: diagnosis with CT and MR imaging. Radiology.

[11] Latgé JP, Chamilos G (2019). Aspergillus fumigatus and Aspergillosis in 2019. Clin Microbiol Rev.

